# Suitability evaluation for mountain-based adventure tourism: A case study of Xinjiang Tianshan, China

**DOI:** 10.1371/journal.pone.0247035

**Published:** 2021-02-23

**Authors:** Cuirong Wang, Zhaoping Yang

**Affiliations:** State Key Laboratory of Desert and Oasis Ecology, Xinjiang Institute of Ecology and Geography, Chinese Academy of Sciences, Urumqi, China; Northeastern University (Shenyang China), CHINA

## Abstract

In recent years, there has been considerable growth in the provision of and demand for adventure tourism; however, research that examines the resources regarding adventure tourism is limited. A spatial suitability evaluation system for mountain-based adventure tourism (MBAT) was developed via the integration of the AHP-Delphi technique. The evaluation system parameters included resource conditions, difficulty levels, safety conditions, and ecological sensitivity. Furthermore, each parameter contained several indicators that can be quantified and visualised in ArcGIS. The results showed that suitable areas for professional adventure tourism in Xinjiang Tianshan include the Kurdening and Tomur regions, and the those for adventure tourism include the Tianshan Tianchi lake. Furthermore, suitable areas for experiential adventure tourism include the Tianshan Tianchi lake, Tianshan Grand Canyon, Jiangbulak, East Tianshan, Tuohurasu scenic area, and the Gongliu wild fruit forest, while those for mass adventure tourism include large areas in the middle and low altitude range of Tianshan. The methods and results proposed in this paper are expected to be significant for planning adventure tourism and can be helpful for mountain communities when choosing regions to develop for adventure tourism, formulating tourism development strategies, increasing tourism opportunities, and thus improving regional competitiveness.

## Introduction

With the increased popularity of traditional sightseeing tourism, the prominent scenic areas tend to be overcrowded, but many tourists remain unsatisfied with only sightseeing. In this context, adventure tourism is widely accepted as a representative practise of eco-tourism. It is one of the fastest growing sectors in the tourism industry, as outdoor recreation has become increasingly commercialised [1].

Adventure tourism originated in "adventure recreation" or "outdoor adventure" [2]. Darst and Armstrong [3] suggested that outdoor adventure provided an instinctive and meaningful human experience, that was directly linked to various unique outdoor environments. Based on this, Yerkes [4] pointed out that adventure activities are outdoor activities dependent upon uncertain conditions. According to Ewert [5], the concept of adventure is an activity that uses the interaction with the natural environment to emphasise the active participation of the individual, that contains a dangerous element, and whose results are uncertain but influenced by the participants and the circumstances prevailing. According to Sung et al. [6], adventure tourism refers to special travel activities that combine risk-taking and manageable risks with personal challenges in the natural or outdoor environment, and the special travel activities are performed in pursuit of new experiences, that suggests that risk factors can be controlled. This implies that adventure activities are not about pursuing serious danger, but rather a manageable risk that can generate excitement among participants. Buckley [7] believes that adventure tourism is a guided business tourism team that primarily attracts natural-based outdoor activities and that excites participants. These activities generally require specialised sports equipment, but the operator of such equipment is not necessarily the tourists themselves. This concept introduces "business" into the definition of adventure tourism; that is, adventure tourism is an organised and guided activity. The organisers of adventure tourism are responsible for the safety of their business. The Adventure Tourism Association [8] decided that tourism that includes at least two of the following elements can be referred to as adventure tourism: 1) interacts with the natural environment, 2) interacts with culture, and 3) requires physical activity. Typical adventure tourism includes these three elements simultaneously [9].

Previous research has focussed on the study of specific adventure activities in specific places. Adventure tourism is generally thought to involve land, air, and water-based activities, ranging from short, adrenalin-fuelled encounters, such as bungee jumping and wind-surfing, to longer experiences, such as cruise expeditions and mountaineering [10]. Hales [11] and Johnson and Godwin [12] reviewed mountaineering and ice climbing around the world, while Hudson [13] studied skiing, heli-skiing, and cross-country skiing. There have also been studies on cross-country mountain biking [14], rafting and kayaking [7], and camel cycling [15]. Buckley [16] systematically studied more than 70 adventure tourism products around the world in terms of price, duration of events, team size, capability, and location of events, and found significant differences between various adventure products, that were distributed from small scale, high difficulty, and high price to large scale, low difficulty, and low price. Water activities such as rafting, kayaking, sea kayaking, and surfing are similar in price each day. Diving and helicopter snowboarding or skiing are particularly expensive owing to special equipment requirements. Based on activity difficulty and team size, Buckley summarises a pyramid model of adventure tourism products. The higher the difficulty level, the smaller the team size, as the tour guide must pay more attention to ensure safety; on the contrary, activities with lower skill or difficulty levels always have a relatively large team size.

Among the different adventure tourism products, mountain-based adventure tourism (MBAT) is an important one. The widely distributed mountain environment provides unique resources for adventure tourism. MBAT has characteristics of both adventure tourism and mountain-based tourism; however, MBAT should be distinguished from mountaineering. Mountaineering generally refers to climbing independent peaks above the snow line (above 5,000 metres in the Tibet Autonomous Region and above 3,500 m in other provinces in China). MBAT includes many activities, such as rock climbing, skiing, hiking, and mountain biking. Mountaineering is a special form of MBAT that is listed at the top of MBAT in terms of technical difficulty, equipment, and funds requirements. By definition, MBAT refers to the commercial tourism activities that people carry out in the mountains with certain risks and challenges.

Different types of MBAT require different mountain resources; therefore, the study of mountain resources is an important prerequisite for the development of MBAT. In addition, unsuitable tourism exploration in a sensitive area will lead to environmental disruption and waste resources [17], thus a resource suitability evaluation is needed to determine the best development model for MBAT. However, the present research is mainly focussed on the qualitative description of MBAT and lacks quantitative analysis of spatial suitability distribution. This study provides a framework for assessing the applicability of MBAT. The approach adopted in this study is a multiple criteria decision analysis (MCDA) integrated in GIS environments, which has been widely used to determine the suitability of fire stations [18–20], ecotourism sites [21,22], campground sites [23], and PV solar plants [24].

This study uses Tianshan Mountain as the study area for investigating the suitability of mountain resources in developing MBAT. Xinjiang Tianshan is a large mountain system located in central Asia that contains many important adventure tourism resources. There are four world natural heritage sites, several famous ancient roads along the historic Silk Road, glaciers, high mountains, forests, valleys, grasslands, lakes, and other natural landscapes as well as rich animal and plant resources. In terms of geological composition, the Tianshan Mountain has a typical geological structure section, stratigraphic section, important biological fossil sites, mineral deposits, natural disaster remains, erosion landscapes, mountain glaciers, and so on. All of the resources mentioned above form valuable mountain adventure tourism opportunities. The methods and results proposed in this paper are of guiding significance for planning adventure tourism and are helpful for mountain communities when choosing appropriate regions for developing adventure tourism products, formulating tourism development strategies, increasing tourism opportunities, and improving regional competitiveness.

## Materials and methods

### Study area

**Fig 1:**
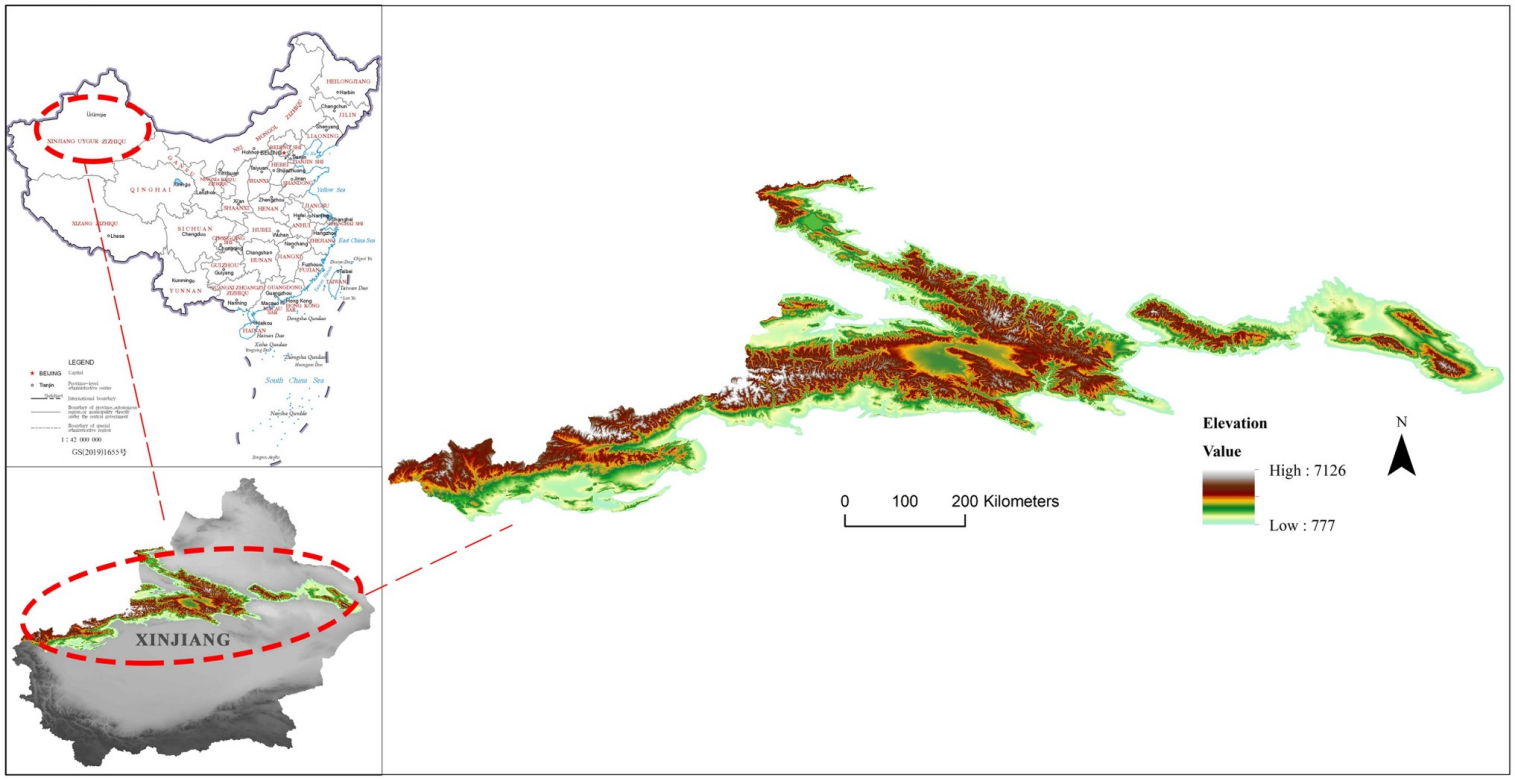
Location of the study area. Visualization based on DEM, which is the digital elevation model from the Shuttle Radar Topography Mission (SRTM) with a 30m spatial resolution downloaded from http://www.gscloud.cn. Republished from http://www.gscloud.cn under a CC BY license, with permission from Geospatial data cloud, original copyright [2020].

Our study area (Fig 1), Xinjiang Tianshan, is approximately 230,000 square kilometres. It is located in the central part of Xinjiang and is approximately 1700 km long. The peak height ranges from 3500–4500 m but can be more than 5000 m at the mountain junction. The Tomur-Khan Tengri mountain knot is the tallest mountain knot in the entire Tianshan Mountains. The snow line is between and 3500–4500 m and many peaks of Xinjiang Tianshan are covered with snow all year. The mountains’ marvellous natural wonders bring together contrasting environments of hot and cold, drought and humidity, and show a unique natural beauty. It also contains the most important habitat for many rare and endangered species and endemic species in Central Asia. To conclude, Xinjiang Tianshan contains world-class mountain adventure tourism resources.

### Data collection

The data used in this research includes Landsat 8 OLI images with a resolution of 90 m from July 2018–2019, 9 0m resolution ASTER GDEMV2 data, 1 km resolution MODIS surface temperature products, road maps, water system maps, land use data, Xinjiang geological maps, administrative division maps, statistical yearbooks, tourism resource data, and other basic geographic information on Xinjiang. Specific information and sources are shown in Table 1. Among them, Landsat 8 OLI images have been systematically corrected for radiation and geometry and can be directly analysed. ENVI 5.1 was used for stitching and band fusion of remote sensing images, and ArcGIS 10.4 was used to stitch DEM data and to unify data projection transformation.

**Table 1:** Data sources.

Data	Source
**Landsat8 OLI**	Geospatial data cloud http://www.gscloud.cn/sources/accessdata/411?pid=263
**GDEMV2 DEM**	Geospatial data cloud http://www.gscloud.cn/sources/accessdata/305?pid=302
**MODIS**	Geospatial data cloud http://www.gscloud.cn/sources/accessdata/343?pid=333
**Water system maps**	National Catalogue Service for Geographic Information http://www.webmap.cn/mapDataAction.do?method=forw&resType=5&storeId=2&storeName=%E5%9B%BD%E5%AE%B6%E5%9F%BA%E7%A1%80%E5%9C%B0%E7%90%86%E4%BF%A1%E6%81%AF%E4%B8%AD%E5%BF%83&fileId=1A5CEBDB34C04A29AAB7E673930498E7
**Road maps**	National Catalogue Service for Geographic Information http://www.webmap.cn/mapDataAction.do?method=forw&resType=5&storeId=2&storeName=%E5%9B%BD%E5%AE%B6%E5%9F%BA%E7%A1%80%E5%9C%B0%E7%90%86%E4%BF%A1%E6%81%AF%E4%B8%AD%E5%BF%83&fileId=1A5CEBDB34C04A29AAB7E673930498E7
**Administrative division maps**	National Catalogue Service for Geographic Information http://www.webmap.cn/mapDataAction.do?method=forw&resType=5&storeId=2&storeName=%E5%9B%BD%E5%AE%B6%E5%9F%BA%E7%A1%80%E5%9C%B0%E7%90%86%E4%BF%A1%E6%81%AF%E4%B8%AD%E5%BF%83&fileId=1A5CEBDB34C04A29AAB7E673930498E7

### Suitability evaluation framework for MBAT

**Fig 2:**
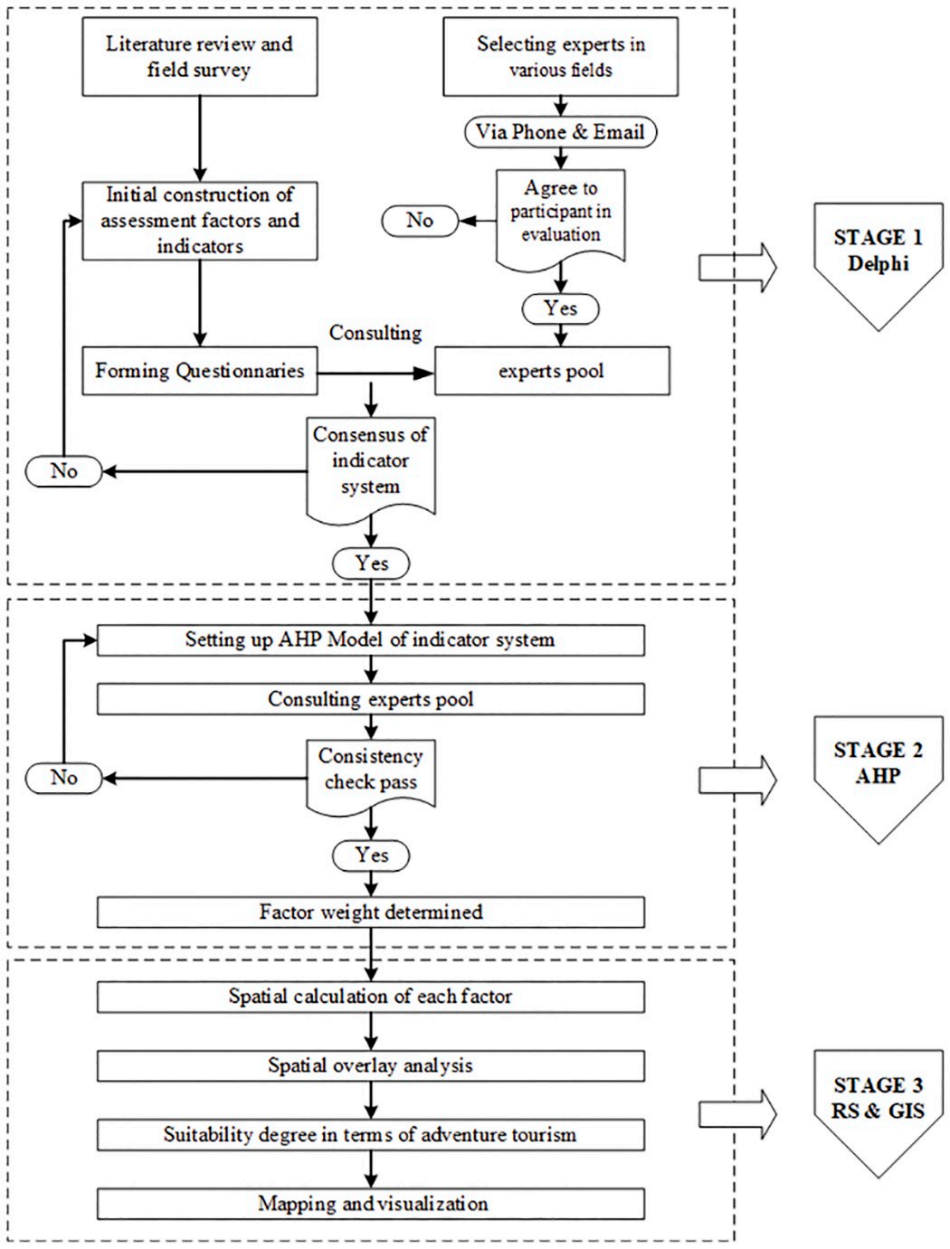
Suitability evaluation framework for MBAT.

This study carried out an evaluation of the suitability of MBAT through three main steps. First, the Delphi method was used to determine the evaluation index system. Second, the AHP was used to determine the index weights. AHP uses the group decision tool in the software yaahp10.1. Finally, ArcGIS 10.4 was used to calculate and visualise the spatial data. The method evaluation framework is shown in Fig 2.

### Determine suitability evaluation index system using Delphi methods

Through the literature review, this study built a suitability evaluation index system including the resource factors, difficulty factors, safety factors, and biological sensitivity factors (Table 2).

**Table 2:** Suitability evaluation index system for MBAT.

Target Layer	Factor Layer	indicator Layer
**MBAT Suitability**	Resources Condition A1	A11 popularity
		A12 aesthetic value
		A13 Annual Suitable Period
		A14 Environmental influence
	Difficulty Condition A2	A21 difference in elevation
		A22 difference in slope
		A23 Remoteness
	Safety Condition A3	A31 Transportation
		A32 Weather Variability
		A33 Geologic hazard
	Biological Sensitivity A4	A41 Elevation
		A42 Slope
		A43 Biodiversity
		A44 Land cover type

Using the Delphi method with expert consultation to screen the evaluation index. We selected experts in various fields who were familiar with our research area. The expert pool included 6 tourism geographers, 3 natural geographers, 4 tourism planning and development experts, 3 adventure tourism organisers, and 4 professional adventure activists. We contacted experts via phone, text message, and email to ask if they were willing to participate in the evaluation: 15 of 20 experts agreed, including 6 tourism geographers, 2 natural geographers, 3 tourism planning and development experts, 2 adventure tourism organisers, and 2 professional adventure activists.

Experts were required to score all indicators with a five-level classification method, with 1 being “least important” and 5 being the “most important.” We collected and analysed the results of the first round of evaluation. Indicators with average scores lower than or equal to 3 were marked. Then the second round of consulting was used to ask the experts whether they agree to remove the marked indicators, and if not, to explain the reasons. We collected and analysed the results of the second round of evaluation, deleted all the indicators agreed by the experts, and conducted a third round of consultation on the indicators at variance with a description of the reasons that some experts stated in the first round of consultation. Note that all expert inquiries were anonymous. After several rounds of consultation, all experts reached a consensus on the indicators, and a total of 14 indicators were included in the evaluation index system.

### Determine indicator weight using AHP

An Analytic Hierarchy Process (AHP) was used to determine the weights of the indexes for the MBAT suitability evaluation. This process was first developed by Saaty in the late 1970s [25] and has been widely used in government, management, engineering, education and other activities [26]. The AHP is a simple and flexible decision-making tool to conduct multi-criteria evaluation [27] and thus can be used with integrated GIS spatial analysis. Five steps must be sequentially performed to output a structured decision using expert judgment through AHP: 1) model the problem as a hierarchy, which was already accomplished in the former part (see Table 2); 2) establish a pairwise comparison matrix to evaluate the priorities among the elements of the hierarchy; 3) synthesize those judgments to yield a set of overall priorities for the hierarchy; check the consistency of the judgments [28]; and reach a final decision based on the results of the process [29].

Establishing the pairwise comparison matrix is the most critical step. Comparison matrix determines the indicator’s relative significance in terms of the particular factor to which it belongs. The form of the comparison matrix is shown in Table 3, where *b*_*ij*_ represents the relative importance of element Bi to Bj for Ak. The relative importance value is generally taken as 1,3,5,7,9, while 2, 4, 6 and 8 represent the median value of adjacent judgments, which can be supplemented when the five levels are not enough. See Table 4 for details of the score.

**Table 3:** Judgment matrix of AHP.

**Ak**	**B1**	**B2**	**…**	**Bn**
**B1**	b11	b12	…	bn1
**B2**	b21	b22	…	bn2
**…**	…	…	…	…
**Bn**	bn1	b2n	…	bnn

**Table 4:** Scale of preferences between two parameters in AHP.

Preference factor	Degree of preference	Explanation
**1**	Equally	Two factors contribute equally to the objective
**3**	Moderately	Experience and judgment slightly to moderately favours one factor over another
**5**	Strongly	Experience and judgment strongly or essentially favours one factor over another
**7**	Very strongly	A factor is strongly favoured over another, and its dominance is shown in practice
**9**	Extremely	The evidence of favouring one factor over another is of the highest degree possible of an affirmation
**2,4,6,8**	Intermediate	Used to represent compromises between the preferences in weights 1, 3, 5, 7 and 9

For any judgment matrix, the following formula should be satisfied:

$$b_{ii}=1$$ (1)

$$b_{ij}=\frac{1}{b_{ji}}$$ (2)

A general consistency of all of the judgments is required, and the index weight will not be reliable as a basis for decision making if the judgments are too inconsistent. To check the consistency of the matrix, the following indexes are calculated:

$$\text{CI}=\frac{\lambda_{max}}{n-1}$$ (3)

$$CR=\frac{CI}{RI}$$ (4)

where CI is the consistency index, λmax is the maximum characteristic root of the judgment matrix, and RI is the average random consistency index (Table 5). CR is the random consistency proportion. When CR equals 0, the judgment matrix has perfect consistency, and the CR value increases as the judgment matrix becomes more inconsistent. The judgment matrix has an acceptable consistency when CR <0.1 [28].

**Table 5:** Numerical value of RI.

N	1	2	3	4	5	6	7	8	9	10
RI	0	0	0.58	0.90	1.12	1.24	1.32	1.41	1.45	1.49

The group decision making tool in Yaahp10.1 was used to assign weights. The judgment matrix aggregation method is adopted to aggregate expert data, that is, according to the expert judgment matrix data, the mean value of each element of each judgment matrix is calculated. After obtaining the mean judgment matrix, the ranking weight is calculated. Results are shown in Table 6.

**Table 6:** Suitability evaluation factor weights of mountain-based adventure tourism.

indicator Layer	Wight
Resource Popularity	0.1409
Aesthetic Value	0.3891
Annual Suitable Period	0.1327
Environmental Influence	0.3373
Difference in Elevation	0.2704
Difference in Slope	0.4839
Remoteness	0.2457
Transportation	0.4079
Weather Variability	0.2614
Geologic Hazard	0.3307
Elevation	0.1631
Slope	0.1872
Biodiversity	0.4269
Land cover type	0.2228

### Data normalization

The spatial data for the 14 indicators used in this study were normalised using reclassification, surface analysis, and buffer analysis in ArcGIS 10.2. The raster data were assigned a designation of 1 (very low) to 5 (very high).

#### Resource popularity

The popularity of tourism resources refers to the degree and scope of tourism resources being understood and recognised by tourists. The more famous the resource is, the more recognised the resource and the more attractive it is to the tourist group. In this paper, the World Heritage Site, a National 5A tourist attraction, National 4A tourist attraction, National 3A tourist attraction, and National 2A tourist attraction are assigned 5, 4, 3, 2, and 1, respectively. The overlapping areas were calculated according to the high grade.

#### Aesthetic value

The aesthetic value of resources is an important factor that attracts tourists. In this study, a viewshed analysis is performed on the main scenic points. The analysis results are divided into five levels according to the natural break classification, and the higher score represents a higher aesthetic value.

#### Annual suitable period

A suitable period refers to the number of days that the scenic spot is suitable to visit. In previous studies, suitable period scores were dependent on expert ratings, which, like popularity, were not suitable for large-scale spatial expression. Therefore, this paper presented the range of suitable periods in space through inversion of MODIS surface temperature products. MODIS can continuously and accurately monitor surface temperatures. The data studied in this paper are MODIS surface temperature products from 2018 to 2019, with a product cycle of 8 d and a spatial resolution of 1 km. The average daily surface temperature of the month was calculated based on the splitting window algorithm. MODIS data were verified according to ground monitoring station data. Finally, 5–1 scores were given by the number of days that average temperature was higher than 18°C longer than 9 months, 7–8 months, 5–6 months, 3–4 months, and less than 3 months, respectively.

#### Environmental influence

Environmental interference has a negative impact on resources. The heavier the trace of human activities, the greater the disturbance to resources. In this paper, through the analysis of buffer zones in villages, towns, and scenic spots, the points less than 1 km, 1–5 km, 5–10 km, 10–20 km, and greater than 20 km are assigned the values 1, 2, 3, 4 and 5, respectively. This index is a reverse index. The higher the score, the lower the degree of environmental disturbance and the better the resource conditions.

#### Difference in elevation

The difference in elevation reflects the ascent. Higher ascents require more from participants’ physical strength and outdoor ability. In this study, the elevation difference is calculated based on the DEM with a resolution of 90 m. We divided the study area into a raster of 1 km by 1 km and calculated the difference in elevation using the formula below:

$$D_{i}=E_{imax}-E_{imin}$$ (5)

where *D*_*i*_ is the elevation difference of the evaluation raster, *and E*_*imax*_ *and E*_*imin*_ are the maximum and minimum values of elevation of the evaluation raster, respectively. According to the natural fracture method, the calculation results were divided into five numerical grades.

#### Difference in slope

The slope difference reflects the roughness of the surface and further reflects the difficulty of the resources to MBAT. In this study, slope difference is calculated based on DEM with a resolution of 90 m and then calculated using "Slope of Slope" (SOS) in ArcGIS 10.2. The results were divided into five grades according to the natural fracture method and assigned 5, 4, 3, 2, or 1 points.

#### Remoteness

Remoteness of the resources determines the load and equipment participants need to prepare and thus reflects the difficulty of MBAT. Based on the existing road network, this paper makes a linear buffer analysis, and gives 5, 4, 3, 2, or 1 points for areas greater than 30 km, 20–30 km, 10–20 km, 5–10 km and less than 5 km, respectively.

#### Transportation

The transportation factor is used to describe the degree of traffic convenience, which determines the timeliness of rescue in case of accident. The higher the level of traffic convenience, the higher the level of safety. In this study, the network density of a road is calculated to illustrate the transportation factor, and the results are divided into 5, 4, 3, 2, and 1 by natural break. The higher the score, the higher the level of traffic convenience and the higher the safety conditions.

#### Weather variability

Unsettled weather is associated with a lower safety level for MBAT. Based on the data of meteorological stations in the study area, the average number of thunderstorms in the scope of a specific county in the last ten years was calculated, and values were assigned spatially and reclassified into five grades, 5, 4, 3, 2, and 1.

#### Geologic hazard

The geological hazards in mountainous areas include earthquakes, mudslides, forest fires, and floods. In this study, the probability of natural disasters is calculated and evaluated through the statistics of geological disasters over the years. Human-caused fires are generally distributed around roads; therefore, this study conducts a linear buffer zone analysis of roads. After calculating the natural and man-made disasters, the space overlay was carried out according to the same weight, and the results were reclassified into five grades using natural break.

#### Elevation

Elevations affect the distribution of flora and fauna and their ability to resist interference as well as the restoration capability after being disturbed. The elevation sensitivity of this study is calculated based on the DEM with a resolution of 90 m. Normally, in mountainous areas, the higher the elevation, the more sensitive the environment is, and the more fragile the ecology is. Thus, elevation was divided into 5 clusters and reclassified into 5, 4, 3, 2, and 1.

#### Slope

The slope describes the steep terrain of the study area. A steeper slope indicates a more sensitive area that is more difficult to restore from human disturbance. In this study, slope calculation was carried out based on DEM with a resolution of 90m, and values of 1, 2, 3, 4, and 5 are, respectively, assigned for slopes between and 0–15°, 16–25°, 26–35°, 36–45°, and above 45°.

#### Biodiversity

In this study, the sensitivity of biodiversity was determined by calculating the proportion of endemic and endangered species in total endemic and endangered species using the formula below.

$$V_{s}=M_{st}/M_{t}$$ (6)

where *V*_*s*_ is the biodiversity index in habitat s, *M*_*st*_ is the number of endemic species and endangered species in habitat s, *M*_*t*_ and is the total number of endemic and endangered species in the study area.

The criteria for selecting endangered species are based on the fauna and flora included in the 1988 State Wildlife under Special Protection, the 2004 Red List of Species, the 2007 Xinjiang Uygur Autonomous Region Wild Plants under Special Protection, the 2010 CITES Appendix and the 2010 IUCN Red List of Species.

#### Land cover type

The sensitivity of different land cover types to human disturbance is different. Based on the 2010 Land use map of Xinjiang, the paper assigns 5, 4, 3, 2, and 1 points to glacier/permanent snow cover area, forest land, grassland, water area, and other/unused land, respectively.

### Assessment of suitability of MBAT

Based on a review of the literature, this article divided MBAT into three categories: skilled adventure tourism, experiential adventure tourism, and mass adventure tourism. The first category, skilled adventure tourism, has the highest requirements for participants’ outdoor skills. Participants enjoy the feeling of challenge and excitement, and the size of the tourist group is generally small. This type of MBAT includes alpine skiing, mountain climbing, rock climbing, and ice climbing. Experiential adventure tourists often participate in adventure activities with the motivation to experience, learn, and enjoy some kind of communion with nature. The risk of experiential adventure is controllable compared to skilled adventure tourism. While skilled adventure tourism is a pure pursuit of challenge and risk-seeking, and aesthetic and scientific values are not the highest priority, experiential adventure has a higher demand for these values in mountain resources. Experiential adventure tourism generally includes multi-day hiking, camping, and wildlife watching. Mass adventure tourism, such as one day hiking, often involves large groups, has a low risk level and does not usually require outdoor skills in the participants. The main motivation of this kind of MBAT is to escape daily routines.

To comprehensively evaluate the suitability of MBAT, various factors need to be integrated. The suitability of each factor is calculated according to the formula below:

$$S_{Ai}=\sum W_{Aij}A_{ij}$$ (7)

where *S*_*Ai*_ is the suitability for factor *A*_*i*_, *W*_*Aij*_ is the weight for indicator *A*_*ij*_, and *A*_*ij*_ is the suitability value of each indicator. Then, the natural break method is used to reclassify each factor to values of 5, 4, 3, 2, and 1. The third step is to divide the suitable areas for different types of MBAT through Delphi. Details of the suitability for MBAT are listed below (Table 7).

**Table 7:** Suitability for different types of mountain-based adventure tourism.

Skilled adventure tourism	Experiential adventure tourism	Mass adventure tourism
Difficulty Condition (A2) > = 3	Resources Condition (A1) > = 3	Difficulty Condition (A2) < = 3
	Difficulty Condition (A2) < = 3	Safety Condition (A3) > = 3
	Safety Condition (A3) > = 3	Biological Sensitivity (A4) < = 3

## Results

### Resources condition

**Fig 3:**
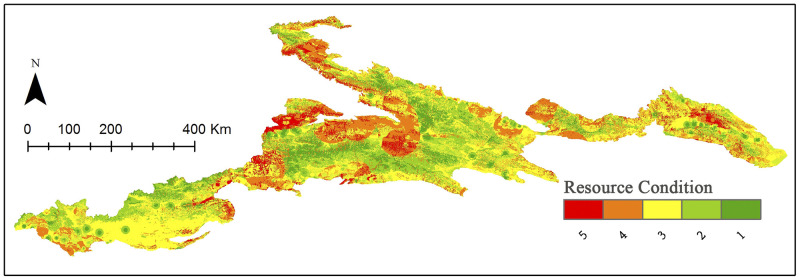
Distribution of the resource classification. Visualization based on data from http://www.gscloud.cn and http://www.webmap.cn/. Republished from http://www.gscloud.cn under a CC BY license, with permission from Geospatial data cloud, original copyright [2020].

Conducting spatial weighted overlay of resource indicators including resource popularity, aesthetic value, annual suitability period, and environmental influence. The spatial distribution of the resource conditions is shown in Fig 3. The results show that Tianshan Mountain has rich adventure tourism resources. Areas of high suitability in terms of resources, i.e. with values higher than 4, account for more than 20% of the total study area. There are four principal components of the Xinjiang Tianshan Natural World Heritage Site: Tomur, Karajun-Kurduning, Bayinbuluk, and Bogda area. Resources with a score of 5 are mostly distributed around the Yili Valley, but some are also distributed in the East Tianshan and Tomur areas.

It can be seen from the statistical results (Table 8) that resources of different values in Tianshan show close to a normal distribution, with resources with 5 scores accounting for 9.78% of an area of about 23,000 km^2^.

**Table 8:** Calculation result of factors classification for MBAT.

Value	Resources		Difficulty		Safety		Biological Sensitivity	
	In km^2^	In %	In km^2^	In %	In km^2^	In %	In km^2^	In %
**5**	22749.92	9.78	17395.92	7.44	36041.73	15.69	26801.06	11.49
**4**	48614.77	20.90	45211.75	19.35	42534.57	18.51	44149.17	18.92
**3**	79379.95	34.12	63286.41	27.08	67525.46	29.39	52690.37	22.59
**2**	56085.96	24.11	66203.80	28.33	50173.95	21.84	66603.29	28.55
**1**	25802.84	11.09	41562.50	17.79	33478.89	14.57	43034.21	18.45

### Difficulty condition

**Fig 4:**
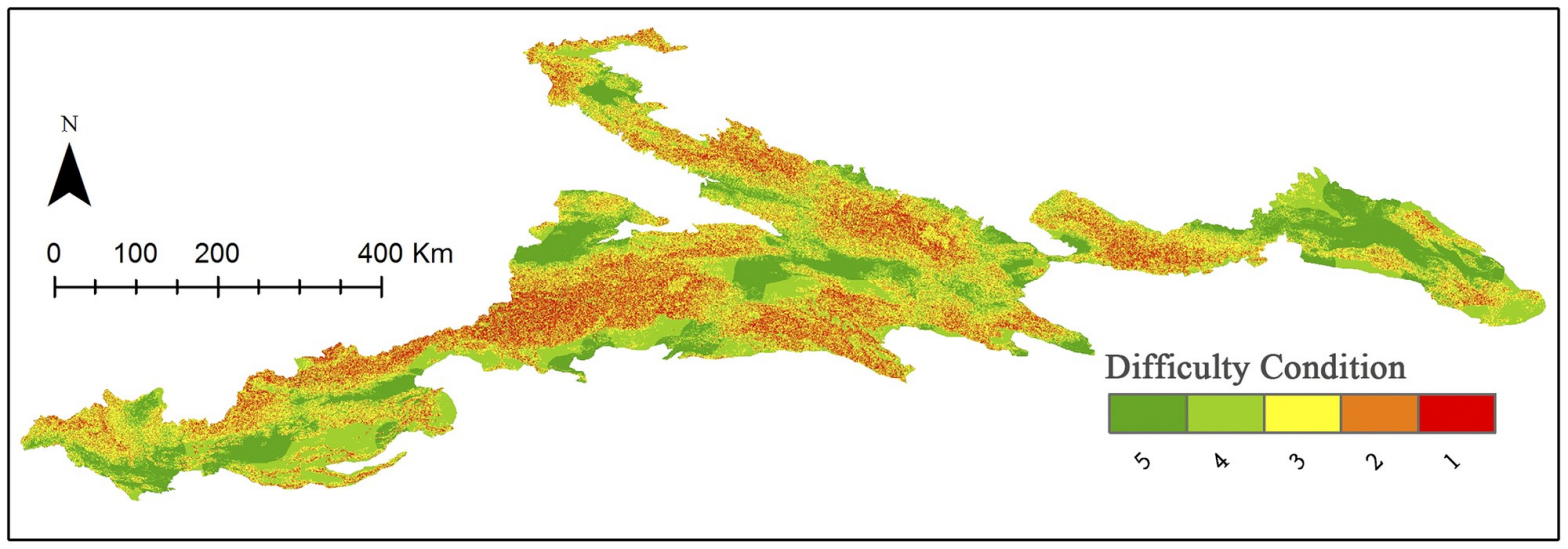
Distribution of the difficulty classification. Visualization based on data from http://www.gscloud.cn and http://www.webmap.cn/. Republished from http://www.gscloud.cn under a CC BY license, with permission from Geospatial data cloud, original copyright [2020].

We conducted a spatial weighted overlay analysis of difficulty indicators including difference in elevation, difference in slope, and difficulty of access. The spatial distribution of difficulty conditions is shown in Fig 4. The results show that the difficulty levels of adventure tourism resources in the Xinjiang Tianshan Mountains are quite varied; however, the distribution at difficulty level 5 is relatively even. The resources at high difficulty levels are distributed throughout the Tianshan Mountains, stretching from East Tianshan to West Tianshan. High-difficulty resources are concentrated in high-altitude areas, with an area of about 17,000 km^2^, accounting for 7.44% of the total area of the Xinjiang Tianshan Mountains (Table 8).

### Safety condition

**Fig 5:**
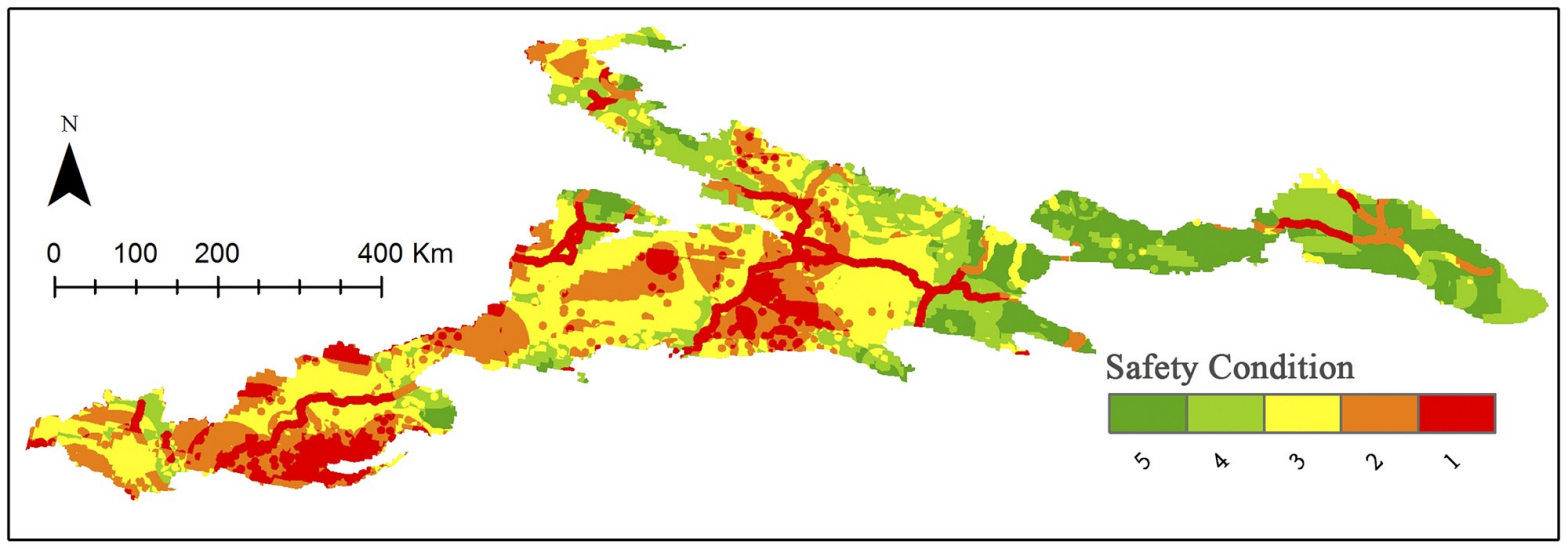
Distribution of the safety classification. Visualization based on data from http://www.gscloud.cn and http://www.webmap.cn/. Republished from http://www.gscloud.cn under a CC BY license, with permission from Geospatial data cloud, original copyright [2020].

Conducting a spatially weighted overlay analysis of safety indicators included considerations of transportation, weather variability, and geological hazards. The spatial distribution of safety conditions is shown in Fig 5. Areas with higher safety levels are concentrated in the eastern part of Tianshan Mountain, while areas with lower security levels are concentrated in the western part. The southern Tianshan Mountains in the Kashgar area tend to have low safety conditions due to frequent earthquakes and frequent thunderstorms. Generally, areas with high security levels (level 4 and level 5) constitute more than 30%, or about 78,000 km^2^ (Table 8).

### Biological sensitivity

**Fig 6:**
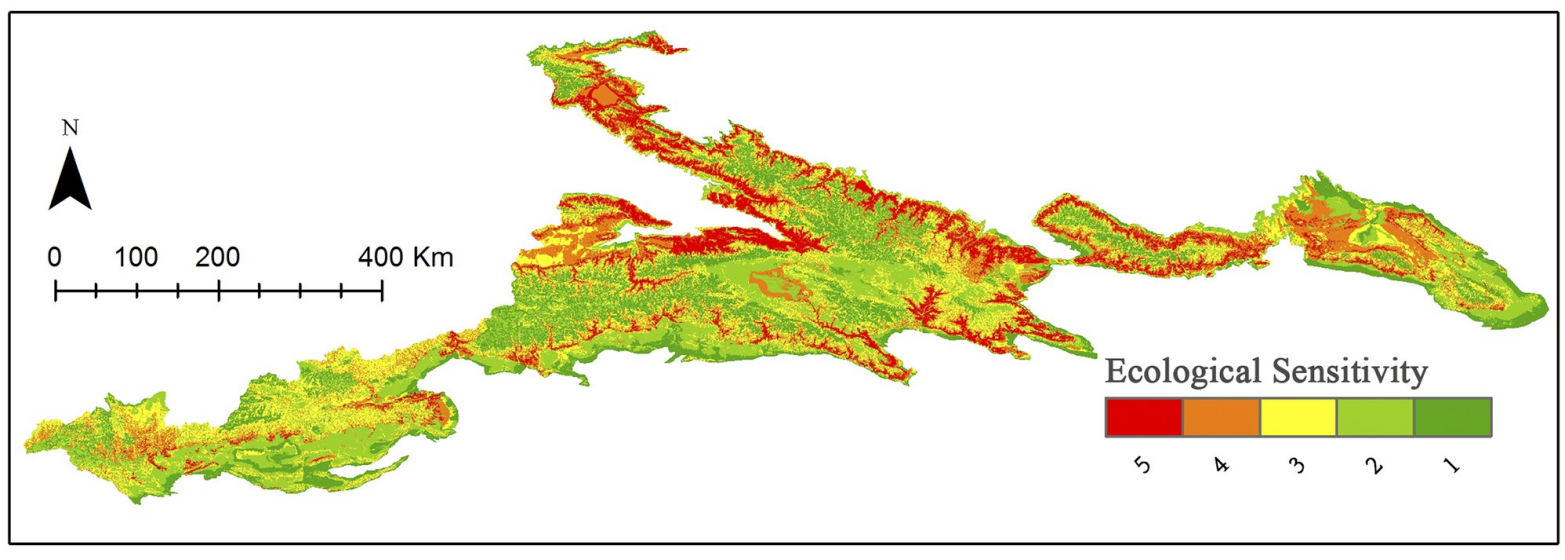
Distribution of the ecological sensitivity classification. Visualization based on data from http://www.gscloud.cn and http://www.webmap.cn/. Republished from http://www.gscloud.cn under a CC BY license, with permission from Geospatial data cloud, original copyright [2020].

We conducted a spatial weighted overlay analysis of biological sensitivity indicators including elevation, slope, biodiversity, and land cover type. The spatial distribution of biological conditions is shown in Fig 6. Areas with high ecological sensitivity in the Tianshan Mountains are primarily distributed in the middle and low altitude areas, largely because the forest habitats of the Tianshan Mountains are distributed in the middle and low mountains-subalpine areas at an altitude of 1500–2800 m. The forest habitat area in the Tianshan Mountains of Xinjiang is rich in biodiverse communities and forms a valuable gene pool for a variety of rare animals and plants. However, the proportion of areas with low ecological sensitivity in the Tianshan Mountains is close to 70% (Table 8), indicating that most areas in the Tianshan Mountains are suitable for adventure tourism from the perspective of ecological sensitivity.

### Resource suitability for MBAT

**Fig 7:**
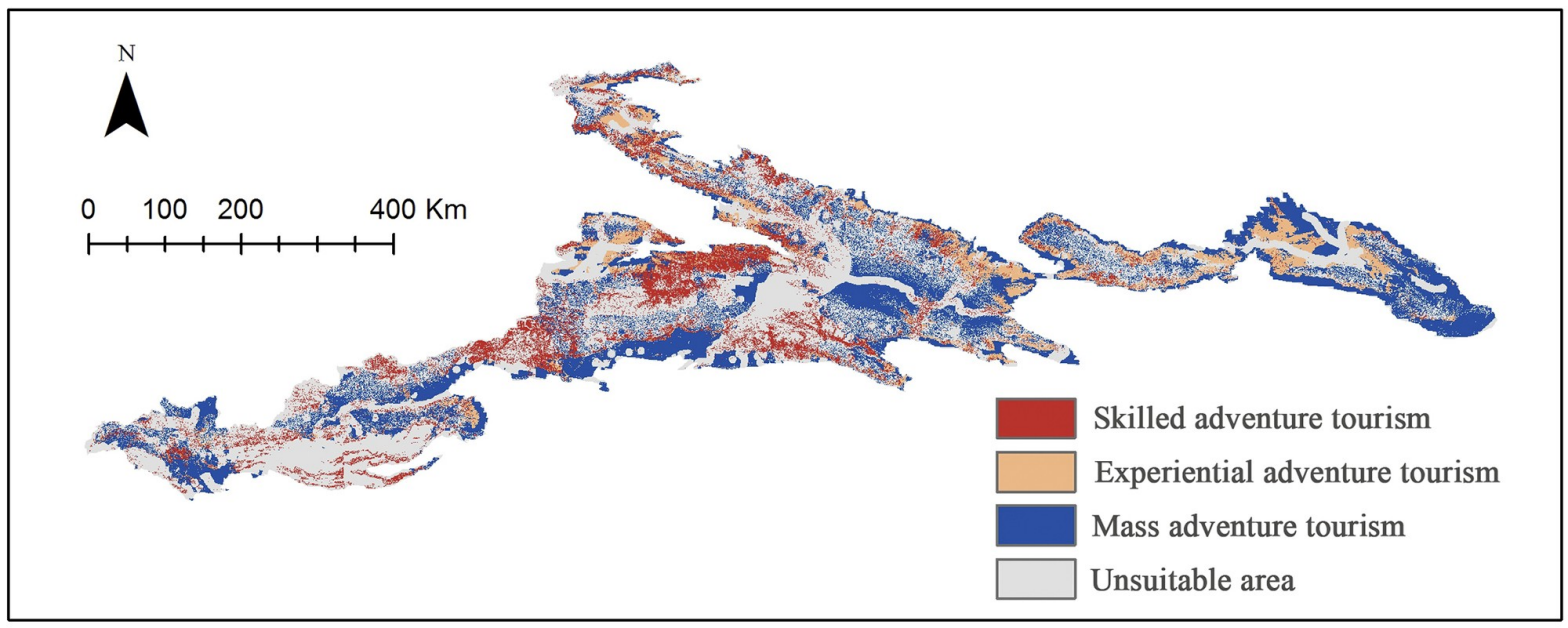
Suitability map for Tianshan mountian-based adventure tourism. Visualization based on data from http://www.gscloud.cn and http://www.webmap.cn/. Republished from http://www.gscloud.cn under a CC BY license, with permission from Geospatial data cloud, original copyright [2020].

Based on Table 7, we used ArcGIS 10.2 to generate suitable areas for skilled adventure tourism, experiential adventure tourism, and mass adventure tourism, respectively (Fig 7, Table 9).

**Table 9:** Calculation result of suitable area for MBAT in Tianshan Moutian.

	Skilled adventure tourism	Experiential adventure tourism	Mass adventure tourism	Unsuitable area
**Area (km**^**2**^**)**	34001.24	22298.54	71600.80	112381.79
**Percentage (%)**	14.1	9.28	29.80	46.77

The results show that most areas of the Tianshan Mountains are suitable for developing different types of MBAT. The unsuitable area is mainly located in the mountain basin at low altitudes. On the one hand, there are many cities and villages in these regions; on the other hand, the lack of undulating terrain makes it impossible to create high-quality adventure tourism resources. Among the three types of MBTA, areas suitable for mass adventure tourism are the most widely distributed (29.8%), followed by skilled adventure tourism (14.1%), and experiential adventure tourism (9.28%). Judging from the conclusion of this article, experiential adventure tourism is the most difficult type of development. This may be because this type of MBAT needs to achieve a balance between adventure and recreation, thrill and safety.

Our results also indicate that suitable areas for professional adventure tourism in Xinjiang Tianshan include the Kurdening Scenic Area, the Tomur Peak Nature Reserve, and the Kuche Grand Canyon. The suitable areas for experiential adventure tourism include Tianshan Tianchi, Tianshan Grand Canyon, Jiangbulak, East Tianshan, the Tuohurasu scenic area, and the Gongliu wild fruit forest; suitable areas for mass adventure tourism include large areas in the middle and low altitude ranges of the Tianshan. Corresponding scenic areas should consider expanding tourism experiences and developing MBAT, such as mountaineering expeditions, canyon crossings, ancient road crossings, alpine skiing, high-altitude heavy hiking, mountain biking, hiking, camping, and rock climbing.

## Discussion and conclusion

The attraction of tourist destinations increases the tourists’ interest in arriving at the destination.

Among the many types of tourism products such as site-seeing tourism, rural tourism, and ice–snow tourism [30], MBAT is a new comer. Mountain tourism destinations are generally located in ecological hot spots, which, on the one hand, have extremely attractive biodiversity resources and landscape resources, and on the other hand, are ecologically sensitive areas, where adventure tourism, such as hiking, are more likely to affected by climate change [31]. At the same time, these areas are also in remote areas, and local economic development is generally relatively stagnant. Protecting the ecological environment while promoting local economic development has always been a crucial issue. As a subset of eco-tourism, adventure tourism can benefit communities located in these mountainous areas. The spending of adventure tourists is also thought to have a far greater impact on the region than that of mass tourists. It is estimated that 5–20% of the consumption by international mass tourists remains in the destination economy, while 65.6% of the income from adventure tourism supports local development [32]. The higher local economic impact is primarily due to adventure tourists being willing to pay more for local guides with activity skills and knowledge for interpretation and safe representation in the local cultural and environmental context. In particular, "more difficult" adventure tourism activities require more skilled tour guides and tour operators, resulting in higher paid jobs and more local economic opportunities [33]. At the same time, adventure tourists have a lower demand for infrastructure, so the negative impact on the environment can be minimised.

Currently, the research on adventure tourism is far behind the development of the adventure tourism industry itself. In fact, the entire tourism research system faces this problem. From the perspective of the tourism industry or from the perspective of tourism social phenomena, academic research on tourism is in an incomplete and unsystematic state [34]. Academia has not formed a basic theoretical system for the research of adventure tourism, nor has it formed a generally accepted and recognized research method.

This article proposes a set of frameworks to evaluate mountain tourism resources for the development of adventure tourism, and it takes Xinjiang Tianshan Mountain as an example to carry out a case study. We built a resource suitability evaluation index system for MBAT. 15 experts in various fields were invited to conduct 3 rounds of consultation through e-mail. Factors of the evaluation system include resource conditions, difficulty levels, safety conditions, and ecological sensitivity. Furthermore, each factor includes several indicators that can be qualified and visualised in ArcGIS. After using the multicriteria overly method, 3 types of MBAT, skilled adventure tourism, experiential adventure tourism, and mass adventure tourism had their own suitability areas.

The results of this research show the immense potential of developing adventure tourism in the Tianshan area, to address the dilemma of resource protection and utilisation by developing adventure tourism. The evaluation results proposed in this research can provide an initial framework for the regional development of adventure tourism. The development of adventure tourism is conducive to enhancing public perception of the region, thus promoting the overall development of regional tourism and driving the development of related industries and economies. Local communities can use customised adventure tourism activities to attract a specific group of people interested in local ecology and culture and engage them in local sustainable development issues.

Currently, there are some problems that are being encountered in the development of adventure tourism in Xinjiang. The first is that local authorities and tourist offices do not attach sufficient importance to adventure tourism. Although ecological protection, economic value, and even dissemination value of adventure tourism are much greater than that of mass tourism, it is still a relatively minor form of tourism, and thus the development of adventure tourism is not fully included in local development planning. Second, although adventure tourism has low infrastructure requirements, the necessary service facilities can greatly reduce the occurrence of accidents. This paper suggests that necessary service facilities should be added to suitable areas for various kinds of adventure tourism in the Tianshan area. For example, environment-friendly road signs should be placed along hiking roads, and mountaineering camps should be built in suitable mountaineering areas. Meanwhile, risk management measures such as insurance, rescue, and supervision should be incorporated into regional development plans.

Limited by the theoretical basis and data access, this research has some shortcomings. The first is that evaluation indicators need to be enriched and improved. The selection of indicators is crucial for the suitability evaluation. The evaluation factors and indicators, as well as weights of indicators in the research, are given by the empirical experiences and personal opinions of experts. Despite using multiple rounds of Delphi, evaluation results are still inevitably influenced by the subjective viewpoints of experts. The second shortcoming is that although this article makes a preliminary suitability assessment of mountain adventure tourism resources, practical development of MBAT still requires more in-depth and meticulous research and more specific and operable implementation planning. Future research on adventure tourism should pay more attention to quantitative and statistical data-based research and should look to the theories and methods of economics, sociology, geography, ecology, and other disciplines to develop multidisciplinary integration.

## Supporting information

S1 File (RAR)
